# General considerations in hypospadias surgery

**DOI:** 10.4103/0970-1591.40614

**Published:** 2008

**Authors:** Amilal Bhat

**Affiliations:** Department of Urology, SP Medical College, Bikaner, Rajasthan, India

**Keywords:** Algorithm, diversion, general considerations, hormonal stimulation, hypospadias, postoperative care, preoperative evaluation, repair, stent, suture material, urethroplasty

## Abstract

Nonsystemic review of the literature was done for timing of surgery, preoperative evaluation and plan, anesthesia, suture materials, magnification, tissue handling, stent and diversion problems, intra and postoperative care, dressing, and follow-up protocol. The best time for hypospadias repair is between 6 and 18 months. Preoperative evaluation in proximal hypospadias includes hormonal and radiological examination for intersex disorders, as well as for upper tract anomalies along with routine evaluation. General anesthesia is a rule but local blocks help in reducing the postoperative pain. Magnification, gentle tissue handling, use of microsurgical instruments, and appropriate-sized stent for adequate period help in improving the results. Hormonal stimulation is useful to improve growth and vascularity of urethral plate and decrease the severity of chordee in poorly developed urethral plate with severe curvature. Urethral plate preservation urethroplasty with spongioplasty is the procedure of choice in both proximal and distal hypospadias. Algorithms are proposed for management of hypospadias both with curvature and without curvature. Two-stage urethroplasty has its own indications. A good surgical outcome may be achieved following basic surgical principles of microsurgery, fine suture materials, choosing one or two-stage repair as appropriate, proper age of surgery, and with good postoperative care. Future of hypospadiology is bright with up coming newer modalities like laser shouldering, robotics, and tissue engineering.

Hypospadiology is still recognized as an expanding and evolving speciality. The results of hypospadias repair have improved in the last three decades. Davis long back said that “I believe the time has arrived to state that the surgical repair of hypospadias is no longer dubious, unreliable, or extremely difficult. If tried and proven methods are scrupulously followed, a good result should be obtained in every case. Anything less than this suggests that the surgeon is not temperamentally fitted for this kind of surgery.”[1] One of John Ducket's many enduring legacies has been the emergence of the ‘hypospadiologist’, i.e., a surgeon committed to excellence in hypospadias surgery with a case load sufficient to develop and maintain a high level of specialist expertise. As a result the era of the ‘occasional’ hypospadias surgeon is fast disappearing. While science undoubtedly merits a higher profile within ‘hypospadiology,’ for the child with this condition what matters most remains the commitment and skill of the surgeon. The traditional saying that “see one, assist one and do one” does not hold true for the hypospadias surgeon; it should be “see many, assist many, do many, and then teach many.” Who should operate the hypospadias? General surgeons, general urologists, pediatric surgeons, pediatirc urologists, or hypospadialogists? As hypospadias surgery is technically demanding, any one of them can, provided the surgeon has a temperament for hypospadias surgery, has mastered six common techniques in hypospadias and has at least 40-50 cases to operate per year. Experience in hypospadias surgery has a definite co-relation with a successful outcome. There is a significant difference in outcome of hypospadias surgery done by pediatric urologists vs. other surgical specialists.[2,3]

John Duckett had said “There are many successful methods, no single procedure works for all hypospadias cases, choose a suitable technique for individual case.”

## TIMING OF SURGERY

Most males with hypospadias are often diagnosed just after birth or identified during examination before a newborn circumcision. Rarely, the ventral foreskin will be normal in appearance and the hypospadias will be noted later in life when the foreskin is retracted or after a circumcision is performed. Treatment starts with birth of the child, the first and foremost step being to inform and console the parents about the congenital anomaly, timing and outcome of surgical procedures, and establishing a bond of confidence between parents and the surgeon. This helps in removing the worry, guilt, and fear of the unknown to parents and in better planning of surgical treatment of the child. Meatal dilatation should be done at the time of first examination if hypospadiac opening is associated with meatal stenosis. The timing of surgery is chosen after considering milestones of development, size of penis, child response to surgery, anesthesia risk, and toilet training. The infant develops good tolerance to surgery and anesthesia by the age of 6 months. The penile length at 1 year is on an average 0.8 cm less than at preschool age. The child is well aware about his genitalia and toilet training by the age of 18 months. So the most suitable age for operation of hypospadias is between 6 and 18 months. Another opportunity is at 3-4 years if the previous optimal age is missed.[4] The American Academy of Pediatrics review suggest that the ideal age for genital surgery is between 6 and 12 months.[5] Others prefer to operate even earlier on an adequate-sized phallus at 4 months of age as healing is quicker with minimal scars and the infant overcomes the stress of surgery easily.[6] Age of presentation (mean age 5 years) to the hospital in the developing countries is higher than in the western part of world because of ignorance, illiteracy, and unaffordability, so patients may be operated whenever the child is brought to the hospital after the age of 4 years.[7]

## PREOPERATIVE EVALUATION

The preoperative assessment includes not only the medical checkup of the child including history of problems, but also counseling of the parents. Parents should be told about the goals of the surgery, plan of surgical repair, likely modifications during surgery, common complications and their treatments, period of hospitalization, postoperative protocol including catheter care, dressings and medications. The perineum is inspected for diaper rash or infection and if present then surgery is to be postponed till such infection is cleared off.

Preoperative examination includes measurement of the size of the penis, shape of the glans, location and size of the meatus, urethral plate for it's development, width and length, severity of hypospadias, length of hypoplastic urethra, chordee and it's severity, size of dorsal hood, shape of the scrotum, and associated anomalies like undescended testis, inguinal hernia or penile torsion. Sometimes multiple pinpoint dimples may be present on the surface of the urethral plate in addition to a hypospadiac meatus and in such cases location of the meatus should be confirmed by a probe.[6] Occasionally probing may confirm the partial duplication of urethra that should be laid open to convert it to one urethra. According to the location of meatus the hypospadias is divided in to anterior (glanular and subcoronal 50%), middle (Distal penile, mid-shaft, and proximal penile 30%) and posterior (Penoscrotal, scrotal and perineal 20%).[8]

Other congenital anomalies associated with severe hypospadias are pelvi ureteric junction obstruction, vesicoureteric reflux, renal agenesis, persistent Mullerian structures and intersex disorders, undescended testis and inguinal hernia with or without hydrocele.[9] Associated anomalies with hypospadias increase with severity of the disease. Patients with severe hypospadias require complete evaluation including ultrasonography for upper tract anomalies and internal sex organs, karyotyping, micturating cystourethrogram and pandoscopy.[4,10] Urethrogram or endoscopy is needed for proper assessment of prostatic utricle which may create problems in catheterization during surgery. Patients with hypospadias of any degree with impalpable one or both gonads should be evaluated for intersex disorders. Such patients should have karyotyping and ultrasonography of the urinary tract and internal genital organs.[10] Accurate assessment of type of hypospadias, severity of curvature, and the urethral quality is often possible under anesthesia and, therefore decision of surgical plan may have to be changed on the table.[4]

## HORMONAL STIMULATION

There is no general agreement on the use of hormonal stimulation in hypospadias surgery. Use of βHCG or testosterone or dihydrotestosterone is sometimes indicated in patients with a small penis or for repeat surgery; it is unclear how safe these treatments are in the longterm.[11] HCG is best suited in cases of patients with undescended testis. But if one suspects a hypogonadotrophic etiological factor of hypospadias then HCG should be used cautiously as experimental micropenis model supports delaying hormonal therapy until puberty.[12]

Local testosterone cream 5% twice a day for 5 weeks is preferred by most of the pediatric urologists and others favour systemic testosterone, as per Koff's regimen (two injections a week for 5 weeks). Hormonal stimulation increases length of penis significantly, increases vascularity and thickness of corpus spongiosum and decreases the severity of hypospadias.[13]

## MAGNIFICATION

Key of success in hypospadias surgery is proper dissection and meticulous approximation of tissues. So magnification becomes an important tool in hypospadias surgery in small children.[11] Various magnification tools are high-powered simple glasses, loupes and operating microscope. The choice depends upon the availability and acclimatization of the surgeon to use the magnification.

## ANESTHESIA

General anesthesia is the rule, often associated with caudal or penile anesthesia. The evidence examined shows an increased duration of analgesia with caudal bupivacaine, clonidine, ketamine and midazolam. However, routine use of these adjuvants in the setting of elective outpatient surgery shows an improved patient outcome. It is unclear if the potential for neurotoxicity is outweighed by clinical benefits. Further testing, including large clinical trials, is required before recommending routine use of nonopioid additives for caudal blockade in children.[14] Routine local penile block at the beginning and ending of surgery significantly improves relief from postoperative pain.[15]

## SUTURE MATERIAL

The composition of suture material and the technique of suture placement may contribute significantly in the outcome of hypospadiac surgery. Significantly low fistula rate (4.95% vs. 16.6%) were noted by Ulman and co-workers in subcuticular repair compared to full thickness through and through technique,[16] while others are of the opinion that sutures used either subcuticular or through and through does not affect the results provided polyglactin suture is used.[17] Late absorbable sutures may be the cause for small fistulae. Usually polyglactin absorbable sutures are useful for the inner most layer closure with epithelial inversion, while polyglyconate sutures are used for other layers.[2] The author is of the opinion that when the technique involves passing the sutures through the epithelium of urethral plate or skin, then early absorbable suture like Vicryl rapid should be used and in subcuticular suturing, any of the absorbable or late absorbable suture material can be used.

## TISSUE HANDLING

General principles in hypospadias surgery include minimal tissue trauma, minimal and pin point use of cautery, tension free repair in all layers, use of well-vascularized tissue closure in as many layers as possible, and single-stage repair with epithelial inversion.[4]

**Figure 1:**
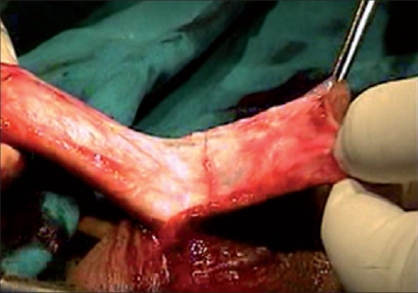
Showing deep plane of dissection at Buck's fascia

**Figure 2:**
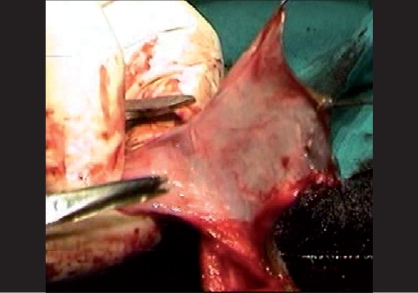
Showing superficial plane of dissection at two layers of dartos fascia

**Figure 3:**
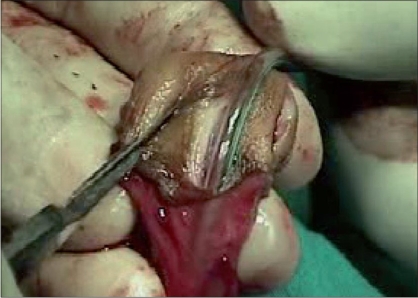
Showing mobilization of corpus spongiosum and urethral plate in to glans

Tissue trauma can be minimized by proper handling of the tissues by using stay sutures, skin hooks, microsurgical instruments and dissecting the tissues in proper plane, maintaining the proper vascularity of the flap for neo-urethra, and skin is very important in prevention of ischemic complications. During penile degloving plane of dissection is kept at the level of Buck's fascia [Figure 1] and while dissecting the inner prepucial flap it is between two layers of Dartos fascia [Figure 2]. For mobilizing the urethral plate and urethra, a plane of dissection is created, beginning at the level of Buck's fascia in normal urethra and then proceeding distally in the same plane. Incision for glanular wings should be in continuity with corpus spongiosum [Figure 3].

## URINARY DIVERSION

Use of stents and diversion is still a debatable issue. In a multicentric retroscopic review of Mathieu's repair, no difference was noted in fistula rate in stented vs. nonstented repair and none of the patients, even in caudal anesthesia group, had urinary retention postoperatively.[17] Others had successful stent free repair with Snodgrass modification.[18] According to some authors, there was significant difference in outcome of stented vs. unstented patients[4] while others claim no difference in outcome.[19] In author's opinion using silastic catheter of adequate size according to the age of child, just inside the bladder for about a week is safer and improves the results. The stent can be left in the diapers and patient can be sent home the same day in day care centers.

## DRESSING

Hypospadias surgeons have different views about postoperative dressings; some concluded that no dressing is required in plate preservation procedures,[19] while others have used various innovative methods. The techniques described and found suitable include polyurethrane bio occlusive foil, Cavi care, SANAV, glove-finger, Fibrin seal (Tisseal), Melolin, Peha-Haft, and adhesive membrane dressings. Silicon foam dressing was found effective in restricting edema, hematoma formation and stabilization with easy removal.[20] Pressure during the dressing following hypospadias repair is a controversial issue. Excessive pressure may compromise the blood supply of flap and skin which may lead to tissue necrosis while no pressure may lead to hematoma, edema and infection increasing the incidences of complications. The author believes that dressing is essential to control postoperative edema, prevent hematoma formation that predisposes to infection and it works as a barrier from surroundings specially in third world countries where the ward cleanliness and hygiene may not be ideal.

## SURGICAL TREATMENT

The goals in management of hypospadias repair are creating a straight penis, reconstructing slit-like meatus at the tip of penis, creating a urethra of adequate length and uniform caliber, symmetry in appearance of glans and penile shaft, projectile stream and normalization of erections, and thereby imposing confidence in the child. These goals can be achieved by meatoplasty and glanuloplasty, orthoplasty, urethroplasty, scrotoplasty and skin cover.

### Meatoplasty and glanuloplasty

**Figure 4:**
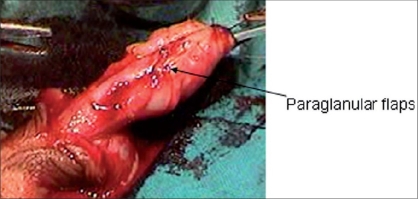
Showing glanular and para-glanular flaps for glanuloplasty

**Figure 5:**
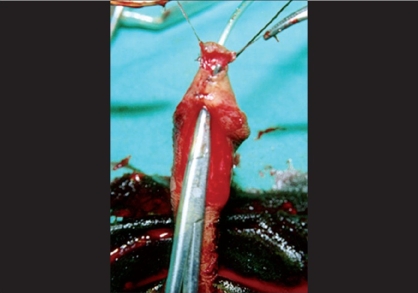
Showing glanuloplasty with adequate space between neourethra and glanular flaps

This helps in creating the conical glans, fish mouth wide meatus at the tip, giving projectile stream and prevents meatal stenosis and fistula. V flap or W shape flap meatoplasty are commonly done with flap urethroplasty. Circumcoronal incision is planned 5-7 mm away from corona to raise para-glanular flaps, suturing of which will reduce the incidence of subcoronal fistula [Figure 4]. An important point in meatoplasty and glanuloplasty is raising adequate length of glanular wing to prevent pressure on the neo-urethra [Figure 5] that will reduce ischemic complications.

### Orthoplasty

Mclaughlin and Gitte's test (1974) is the most important mile stone in deciding the single-stage repair. Straight penis is the first requirement for a successful repair and complete chordee correction, should be tested on table before proceeding for the urethroplasty. Various method of chordee correction are penile de-gloving, plication procedures, split and roll technique, extended urethral mobilization, penile disassembly and tunica grafting procedures. Rational approach in correction of chordee is described step-by-step by Bhat 2007[7] and Bhat *et al.* 2007.[21] Pharmacological erection by intracorporeal injection of Prostaglandin E1 has been found useful both intraoperative as well as in follow-up visits to check for correction of curvature.[22]

### Urethroplasty

Various factors in deciding the type of urethroplasty are size of penis, chordee, location of the meatus, size and configuration of the glans, development and width of urethral plate, development of corpus spongiosum, length of hypoplastic urethra, ventral penile skin proximal to the meatus and skin available on the dorsal hood and penile shaft.

**Figure 6:**
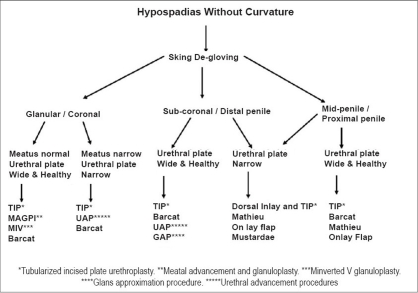
Algorithm 1 for hypospadias without curvature

**Figure 7:**
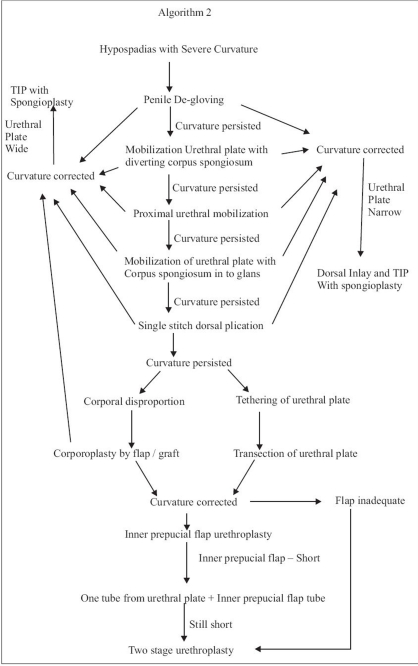
Algorithm 2 for hypospadias with curvature

Urethral plate preservation procedures are preferred as there is no substitute for urethra. Final decision about the type of urethroplasty is to be taken only after correction of chordee. In distal and middle hypospadias with minimal chordee or without chordee, the first choice of procedure is TIP urethroplasty with inlay graft if required and second is onlay flap urethroplasty. An algorithm [Figure 6] is proposed for choice of procedures in hypospadias without curvature. The controversy still continues in management of proximal hypospadias. On one end of the spectrum are one-stage procedures with utilization of urethral plate[7,23,24] and on the other are two-stage procedures.[2,25,26] Lam *et al.* reported spraying of stream in 40%, milking of urethra after voiding in 40%, milking of the ejaculate 42.9%, and painful ejaculation in 7.7% in spite of good cosmetic results in two-stage procedures.[27] So hypospadias surgery should aim to preserve and utilize the urethral plate and supplement with spongioplasty to improve the results. To avoid the confusion, a rational approach is proposed [Algorithm 2, Figure 7] taking into consideration all the factors influencing the repair with stress on preserving the urethral plate and one-stage urethroplasty.[7,21,28]

### Healthy tissue cover

**Figure 8:**
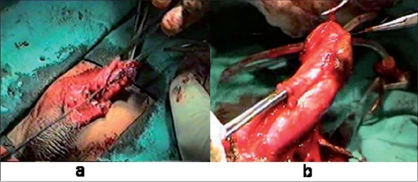
(a) and (b) Showing dartos flap as healthy tissue cover

**Figure 9:**
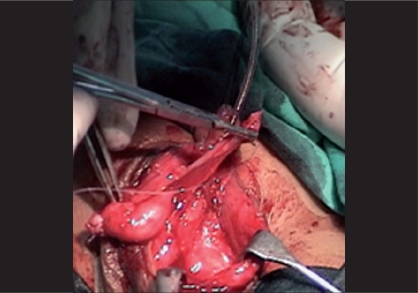
Showing tunica flap as healthy tissue cover

**Figure 10:**
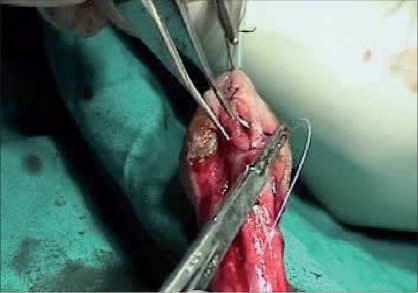
Showing spongioplasty as healthy tissue cover

Healthy-vascularized tissue cover over neourethra or corporal graft definitely helps in overall surgical success. Poorer the tissue more is the need to provide healthy vascularized tissue to optimize the chances of success. Various healthy and well-vascularized tissues used are dorsal/ventral dartos flap, [Figure 8] tunica vaginalis, [Figure 9] denuded inner prepucial skin, and spread out corpus spongiosum [Figure 10]. Dorsal dartos vascular pedicle is mobilized up to root of penis to avoid torsion and tunica vaginalis requires adequate mobilization on its vascular pedicle to prevent inherent sequelae of torque. Skin is to be denuded completely to prevent the complication of buried skin inclusion dermoid. Spongioplasty is the most suitable healthy tissue cover for neourethra and reconstructs a near normal urethra.

## PREPUTIOPLASTY

In distal hypospadias, surgery is done more for cosmetic appearance. Many parents and patients demand prepucial reconstruction. Circumcision is less acceptable to both the general population and medical profession; the prepuce can be preserved and refashioned to give good cosmetic results.[29] Preputioplasty is feasible in patients where prepuce is not utilized in urethroplasty and satisfies the patients and parents to have an uncircumcised penis. This adds about 20 extra minutes to the operating time. Klijn *et al.* had higher complications of urethroplasty with preputioplasty and they discouraged preputioplasty when circumcision is done.[30] While others recommend preputioplasty since they had no difference in results with preputioplasty.[29,31,32] Author is of the opinion that preputioplasty is to be added to urethroplasty in distal hypospadias where prepuce is not utilized and parents demand preservation of prepuce.

## SKIN COVER

Dorsal hood is brought to ventrum by Byar's technique or by Nesbit's technique. Disadvantage of Nesbit is the suture line on lateral side, but it helps in reducing the fistula rate. Author recommends the midline suture simulating median raphae with trimming of skin margins to avoid ischemic complications. In two-stage procedures or in redo cases, there may be tension on suture line requiring dorsal releasing incision or some times nongenital skin graft is needed.

## POSTOPERATIVE CARE

The important points which require attention in postoperative period are dressing, catheter care, analgesics and antibiotics. Postoperatively the child may experience incisional pain and pain related to bladder spasms. We treat incisional pain with acetaminophen or acetaminophen with codeine. Bladder spasms are best treated acutely with Oxybutynin (0.2 mg/kg/dose every 6 h). Prophylactic antibiotics are advised till patient is on catheter drainage. The parents are instructed to apply an antibiotic ointment to the tip of the glans penis and urethra meatus every time the diapers are changed or after passing urine.

## FOLLOW-UP PTOTOCOL

Usual period of follow-up is for two years after surgery as it is expected that by this time most of the complications will appear and follow-up beyond the period may not be cost effective. Any patient with complications beyond this period will automatically present to the surgeon. Early discharge is being justified on the grounds that it is best to let the patient forget his genital abnormality and surgery. Repeated visits to the hospital will remind the child of his abnormality and may have psychological implications. Only a section of surgeons like adult urologists have an access to these patients up to teenage and may contribute a lot in long-term follow-up, actual outcome of surgery and real incidence of chronic complications. An ideal follow-up will be at 1, 3, 6 months and then yearly up to 2 years and review follow-up at puberty and mid-teens by which time genital maturity is at or near completion and patient can express his social and sexual problems following genital surgery. A previous asymptomatic fistula too may start leaking, chordee may appear due to failure of growth of scarred urethra, shape and size of the penis may be of concern to the patient. These late complications may need to be treated.

## FUTURE

Though many new concepts and innovations have been added, but the importance of current techniques are not going to be obsolete suddenly. Future of hypospadiology is directed toward the new innovative techniques and use of developments in biotechnology like LASER shouldering, robotics, and tissue engineering. Laser shouldering has been tried, but it still has to prove its results better than conventional suturing. Urethral regeneration has been identified as one of many potential applications of tissue engineering. Tubular acellular collagen matrices seeded with urothelial cells have been used experimentally with success to repair a created urethral defects in a rabbit model. Similarly others have grown and used the corpus cavernosum.[33,34] But the complexity of urethral structure (urethral mucosa surrounded by spongiosum) makes it difficult to do urethral replacement by tissue engineering. So it is unlikely that there will be routine use of the technology in hypospadias surgery, though it could find a limited role in complex salvage cases. Robotics can play a major role by removing the effects of tremors for meticulous suturing.
